# Terapia de Anticoagulação em Pacientes com Fibrilação Atrial não Valvar em Ambiente de Cuidado de Saúde Privado no Brasil: Um Estudo de Mundo Real

**DOI:** 10.36660/abc.20180076

**Published:** 2020-04-06

**Authors:** Pedro Gabriel Melo de Barros e Silva, Henry Sznejder, Rafael Vasconcellos, Georgette M. Charles, Hugo Tannus F. Mendonca-Filho, Jack Mardekian, Rodrigo Nascimento, Stephen Dukacz, Manuela Di Fusco

**Affiliations:** 1 Hospital Samaritano Paulista São PauloSP Brasil Hospital Samaritano Paulista, São Paulo, SP – Brasil; 2 United Health Group-Brasil Rio de JaneiroRJ Brasil United Health Group-Brasil, Rio de Janeiro, RJ – Brasil; 3 United Health Group MinnetonkaMinnesota EUA United Health Group, Minnetonka, Minnesota – EUA; 4 Optum Life Sciences Eden PrairieMinnesota EUA Optum Life Sciences, Eden Prairie, Minnesota – EUA; 5 Pfizer Inc. New York EUA Pfizer Inc., New York – EUA; 6 Pfizer Inc. São PauloSP Brasil Pfizer Inc., São Paulo, SP – Brasil

**Keywords:** Varfarina/uso terapêutico, Anticoagulantes/uso terapêutico, Antcoagulantes/efeitos adversos, Fibrilação Atrial/complicações, Hospitais Privados/economia, Qualidade, Acesso e Avaliação de Assistência à Saúde

## Abstract

**Fundamento:**

A segurança e a eficácia da varfarina dependem da qualidade do controle da anticoagulação. Estudos observacionais associam controle deficiente com aumento de morbidade, mortalidade e custos com saúde.

**Objetivos:**

Desenvolver um perfil de pacientes com fibrilação atrial não valvar (FANV) tratados com varfarina em ambiente ambulatorial e hospitalar privado brasileiro, avaliar a qualidade do controle da anticoagulação e sua associação com resultados clínicos e econômicos.

**Métodos:**

Este estudo retrospectivo, por meio de um conjunto de dados de seguros privados de saúde no Brasil, identificou pacientes com FANV tratados com varfarina entre 01 de maio de 2014 a 30 de abril de 2016, descreveu seu manejo da anticoagulação e quantificou os custos relacionados à doença. Foram recuperados dados demográficos, histórico clínico, medicação concomitante e tempo na faixa terapêutica (TTR) dos valores da razão normalizada internacional (RNI). Os pacientes foram agrupados em quartis de TTR, com um bom controle sendo definido como TTR ≥65% (método de Rosendaal). Sangramentos maiores e custos médicos diretos por todas as causas foram calculados e comparados entre subgrupos de controle bons e ruins. Valores de p < 0,05 foram considerados estatisticamente significantes.

**Resultados:**

A análise incluiu 1220 pacientes (mediana de seguimento: 1,5 anos; IIQ: 0,5–2,0). Em média, cada paciente recebeu 0,95 medidas mensais de RNI (RNI média: 2,60 ± 0,88, com 26,1% dos valores < 2 e 24,8% > 3), (mediana de TTR: 58%; IIQ: 47-68%), (TTR médio: 56,6% ± 18,9%). Apenas 31% dos pacientes estavam bem controlados (TTR médio: 78% ± 10%), com 1,6% apresentando grandes sangramentos na mediana do seguimento e custos médicos diretos por membro por ano (PMPY) de R$25.352 (± R$37.762). Pacientes com controle abaixo do ideal (69%) foram associados a 3,3 vezes mais sangramentos graves (5,3% *vs.* 1,6%; p <0,01) e custos 40% maiores (R$35.384 *vs.* R$25.352; p < 0,01).

**Conclusões:**

Mais de 60% dos pacientes estavam abaixo da meta desejada e os custos associados foram significativamente maiores nesta população.

## Introdução

A fibrilação atrial (FA) é a arritmia cardíaca sustentada mais comum que afeta mais de 33 milhões de pessoas em todo o mundo. A maioria dos casos é de pacientes com FA não valvar (FANV).^1^^-^^3^ Os dados epidemiológicos da FA na América Latina são limitados, e uma proporção significativa dos pacientes tem pouco controle dos principais fatores de risco e não recebe tratamento apropriado com anticoagulante (18,3 % - 24,6%).^4^^,^^5^

As diretrizes clínicas recomendam o uso de um anticoagulante oral (ACO) na FANV para reduzir o risco de acidente vascular cerebral.^2^^,^^3^ Durante décadas, os antagonistas da vitamina K (AVK) têm sido a pedra angular da terapia com ACO na FANV, sendo a varfarina a mais utilizada dentre os antagonistas. No entanto, a segurança e a eficácia da varfarina têm limitações, e dependem da alta qualidade do controle da anticoagulação.^2^ Isso é conseguido utilizando-se uma medida padronizada do tempo de coagulação, conhecida como razão normalizada internacional (RNI), e cujo valor desejado está entre 2 e 3.^6^ É necessária uma monitorização frequente da RNI e ajuste da dose para manter os níveis alvo da RNI.^2^^,^^3^ No entanto, a monitorização pode aumentar os custos médicos e econômicos.^7^

O tempo na faixa terapêutica (TTR, *Time in Therapeutic Range*) é o meio padrão de avaliar a qualidade em longo prazo do controle da anticoagulação e o perfil de risco-benefício da varfarina.^6^ O TTR representa a porcentagem de tempo em que os valores da RNI de um paciente ficam entre 2 e 3, obtendo o máximo benefício quando o TTR é de 60%-70% ou mais.^2^ Na América Latina, a mediana do TTR estava na extremidade inferior dos níveis recomendados de anticoagulação (cerca de 60%).^4^^,^^8^ No Brasil, alguns estudos observacionais no ambiente hospitalar público mostrou que a maioria dos pacientes apresentava um bom controle de anticoagulação, embora os níveis de TTR estivessem na extremidade inferior do limiar.^9^^-^^11^ O manejo do uso de ACO, incluindo o monitoramento da RNI, é caro e inacessível para muitos pacientes na América Latina.^4^ Até o momento, poucos estudos foram realizados em ambientes hospitalares privados na região. Associações de níveis de TTR com resultados clínicos ou econômicos em geral não foram relatadas. O objetivo deste estudo foi desenvolver um perfil de pacientes recebendo varfarina para FANV em ambientes hospitalares privados no Brasil e avaliar a qualidade do controle da anticoagulação e os desfechos clínicos/econômicos.

## Métodos

### Fontes de dados

Os dados de 1º de maio de 2014 a 30 de abril de 2016 foram extraídos de um grande conjunto de dados de um seguro privado de saúde no Brasil – AMIL. A AMIL é uma das maiores empresas de seguros de saúde do Brasil, com cerca de 4 milhões de beneficiários e programas de assistência clínica com informações integradas e estruturadas sobre doenças prevalentes. O conjunto de dados da AMIL combina registros médicos eletrônicos, contendo informações sobre dados demográficos, registro e histórico clínico dos pacientes, com solicitações médicas de atendimentos ambulatoriais e internações hospitalares, instalações de atendimento ambulatorial e departamentos de emergência.

Para pacientes tratados com varfarina, a AMIL administra um programa privado, por telefone, de monitoramento de anticoagulação denominado VIVA AMIL.^12^ Nesse programa, enfermeiros e técnicos de enfermagem treinados realizam chamadas telefônicas mensais para os pacientes para coleta de dados, resultados autorrelatados do último teste de RNI, ocorrência de eventos tromboembólicos e hemorrágicos, regularidade da medicação e efeitos adversos.

Um modelo existente, criado para capturar dados dos pacientes monitorados, foi utilizado para garantir que os resultados dos testes e os eventos e padrões experimentados fossem relatados de forma consistente para atender às necessidades do programa. Foi feita uma chamada inicial para coletar dados clínicos e demográficos (se não estivessem disponíveis), incluindo a presença de condições crônicas e medicamentos em uso. Cada paciente então recebia chamadas mensais, mas também tinha a opção de ligar sempre que necessário.

Caso o paciente não possuísse um resultado atual ou recente de RNI, um(a) enfermeiro(a) forneceria o suporte solicitando o teste e lembrando-o de ligar e relatar os resultados. Quando os resultados de RNI relatados pelo paciente estavam fora da faixa alvo (RNI 2-3), o(a) enfermeiro(a) discutia os ajustes da dose com o paciente e o aconselhava a procurar um médico.

### Seleção de Pacientes

Os pacientes com 18 anos ou mais foram incluídos caso apresentassem diagnóstico de FA (CID-10-CM código I48) ou fossem avaliados para FA em um formulário específico do sistema no prontuário eletrônico, se tivessem recebido pelo menos uma prescrição de AVK durante o estudo, tivessem cobertura contínua do plano de saúde e se fossem acompanhados pelo programa de monitoramento por telefone por pelo menos 4 meses, com um registro das ligações em pelo menos 50% dos meses durante o período do estudo. Foram excluídos pacientes com evidência de estenose mitral moderada/grave, tromboembolismo venoso (TEV) ou válvula protética mecânica. O protocolo de pesquisa foi aprovado pelo Conselho de Ética Institucional local.

### Variáveis e medidas de desfechos

As principais características dos pacientes que receberam varfarina foram analisadas a partir de solicitações, registros médicos eletrônicos e autorrelatos: dados demográficos e histórico clínico (escore CHA_2_DS_2_-VASc, comorbidades, AVC ou sangramento prévio, RNI e TTR). Especificamente, os pacientes foram classificados como tendo insuficiência renal crônica quando havia pelo menos um dos códigos selecionados da CID-10 (Apêndice A) vinculados a eles no conjunto de dados durante todo o período do estudo, ou se houvesse insuficiência renal crônica no formulário de coleta de dados manejado pelo(a) enfermeiro(a). A utilização concomitante de medicamentos e os padrões de frequência da RNI também foram avaliados.

Consistente com as diretrizes e estudos anteriores,^2^^,^^6^ a qualidade do controle da RNI foi baseada na porcentagem de tempo durante o qual um paciente que recebeu warfarina esteve dentro da faixa terapêutica (2,0-3,0) durante todo o período de seguimento. Um controle bom foi definido como TTR ≥ 65%. O número de testes de RNI para cada paciente foi obtido através do conjunto de dados de solicitações, o qual não registrou os valores de RNI. Durante as chamadas de monitoramento telefônico, o(a) enfermeiro(a) com treinamento solicitava ao paciente que informasse os valores dos testes RNI realizados desde a última chamada. A frequência do teste de RNI foi utilizada para calcular o número das RNI total e média por paciente. Como a RNI é um procedimento de baixa complexidade e baixo custo, o teste poderia ter sido pago diretamente pelo paciente e, portanto, não ter sido relatado nas solicitações. Para reduzir o impacto dos testes RNI não relatados, durante as chamadas de monitoramento telefônico o(a) enfermeiro(a) solicitava ao paciente que informasse também a data do teste de RNI, juntamente com os valores de RNI. Nos casos em que uma solicitação correspondente estava ausente, as informações de frequência do teste no prontuário eletrônico eram adicionadas manualmente pelo(a) enfermeiro(a). A TTR foi calculada pelo método de Rosendaal, calculado com os valores de RNI registrados nos prontuários eletrônicos.^13^

Os desfechos clínicos avaliados foram eventos hemorrágicos maiores e menores, identificados utilizando os códigos da CID-10 de solicitações de pacientes internados, listados no Apêndice A.^14^ Também foram consideradas situações autorreferidas. Os códigos de diagnóstico utilizados para sangramentos maiores foram baseados em um algoritmo administrativo validado baseado em solicitações, bem como na definição da *International Society on Thrombosis and Hemostasis* de sangramentos maiores.^15^^,^^16^ As taxas de sangramento foram calculadas como o número de pacientes com pelo menos um autorrelato de episódio de sangramento durante o período de monitoramento dividido pelo número total de pacientes. Para avaliar os desfechos, os pacientes foram acompanhados até 30 de abril de 2016, a menos que a interrupção do plano de saúde ou a morte ocorresse primeiro.

Os custos médicos diretos por todas as causas foram avaliados a partir das solicitações de cada paciente para consultas eletivas em consultório, atendimentos de emergência, testes/procedimentos ambulatoriais, internações e transição para tratamento/assistência domiciliar. Os custos representaram os custos reais pagos pelo provedor de seguros (AMIL). Os custos desembolsados pelos pacientes não foram incluídos. Os custos estavam disponíveis na base de dados durante o período do estudo e foram anualizados dividindo-os pelos meses do período do estudo e multiplicando-os por 12. Após esse cálculo, os custos foram expressos por membro por ano (PMPY, *per member per year*), em reais (R$) e convertido para dólares americanos. Foi obtido um fator de conversão de 0,33 USD/BRL pela média das taxas de câmbio diárias em cada ano do período do estudo (1º de maio de 2014 a 30 de abril de 2016). As taxas de câmbio diárias foram obtidas dos registros históricos de uma calculadora pública de conversão de moeda.^17^

Os custos bancados pelos pacientes não foram incluídos. Finalmente, as características-chave, resultados clínicos e econômicos foram observados e comparados entre os quartis de TTR.

### Métodos estatísticos

Devido à natureza exploratória do estudo, as principais características e desfechos foram analisados descritivamente.

As estatísticas descritivas foram relatadas como contagens, porcentagens, médias, medianas, desvios-padrão e quartis. As variáveis contínuas foram descritas como média e desvio padrão ou mediana e respectivo intervalo interquartil, dependendo se uma distribuição normal foi ou não encontrada. As variáveis categóricas foram descritas como frequências e porcentagens. As comparações foram feitas entre variáveis contínuas utilizando um teste *t* de duas amostras independentes e não pareadas e entre variáveis categóricas através do teste do qui-quadrado. Valores de p < 0,05 nos testes bicaudais foram considerados estatisticamente significantes. Todas as análises foram realizadas utilizando-se o *software* SAS 9.4.

### Análise de subgrupos

As principais características, os resultados clínicos e econômicos foram analisados para a população em geral e para pacientes com controle ruim (TTR < 65%) e bom (TTR ≥ 65%).

### Análise de sensibilidade

Para verificar a consistência da análise principal, algumas características dos pacientes – níveis de RNI, TTR e custos PMPY – foram observadas para um grupo de pacientes acompanhados por pelo menos 6 meses, com registros das chamadas em pelo menos 50% dos meses durante o período do estudo.

## Resultados

### Características dos pacientes

**Figura 1:**
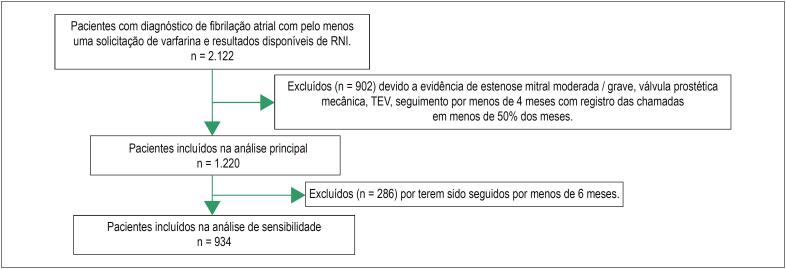
Fluxograma descrevendo os critérios de inclusão e exclusão.

Um total de 1.220 pacientes com FANV foram incluídos na análise principal (Figura 1). No geral, o seguimento médio foi de 1,5 anos (intervalo interquartil [IIQ]: 0,5–2,0 anos). As principais características dos pacientes estão listadas na Tabela 1. A média de idade foi de 63,9 ± 14,7 anos, e 50,7% eram do sexo feminino. A escore médio no CHA_2_DS_2_-VASc foi de 2,45 ± 0,88. A maioria dos pacientes (85,7%) era da região sudeste do Brasil. Aproximadamente 10% dos pacientes estavam em terapia concomitante com estatina e uma parcela menor dos pacientes (~ 4%) estava recebendo terapia concomitante de antiagregantes plaquetários com aspirina e/ou Clopidogrel. A hipertensão foi a comorbidade mais prevalente (38,5%), seguida por insuficiência cardíaca (19,8%), acidente vascular cerebral anterior (13,7%) e diabetes (13,6%).

**Tabela 1:** Características e dados dos pacientes por quartil de TTR

Valores	Período (meses)					
			4 – 24			6 – 24 (Análise de sensibilidade)
	Q1 N = 303	Q2 N = 306	Q3 N = 305	Q4 N = 306	Total N = 1220	N = 934
**Dados demográficos**						
Idade (média/±DP)	62,02 (±15,92)	64,58 (±13,83)	64,49 (±14,48)	64,30 (±14,63)	63,85 (±14,75)	64,75 (±14,03)
Feminino (%)	50,8	55,2	52,5	44,1	50,7	51,5
**Anti-coagulação**						
RNI (média/±DP)	2,56 (± 1,25)	2,67 (± 1,10)	2,61 (± 0,89)	2,54 (± 0,62)	2,60 (±0,88)	2,60 (±0,96)
RNI (mediana/IIQ)	2,22 (1,70-3,16)	2,50 (1,97-3,20)	2,48 (2,06-2,98)	2,44 (2,13-2,78)	2,44 (1,99-3,00)	2,43 (2,00-3,00)
TTR (média/±DP)	32,6% (±11,5%)	51,2% (±3,3%)	62,0% (±3,2%)	80,2% (±9,8%)	56,6% (±18,9%)	58,0% (±16,2%)
TTR (mediana/IIQ)	36% (28-42%)	52% (48-54%)	62% (59-65%)	78% (72-86%)	58% (47-68%)	57,0% (45-68%)
Testes INR por paciente (média/±DP)	12,79 (±8,09)	17,49 (±9,65)	18,00 (±9,27)	14,20 (±8,40)	15,63 (±9,13)	18,44 (±8,60)
**Fatores de risco e condições basais**						
CHA_2_DS_2_-VASc (média/±DP)	2,38 (±1,72)	2,46 (±1,69)	2,55 (±1,69)	2,44 (±1,74)	2,45 (±1,71)	2,58 (±1,72)
AVC (%)	13,9	12,1	11,5	17,3	13,7	14,6
Hipertensão (%)	33,3	39,2	43,6	37,9	38,5	41,3
Diabetes (%)	13,2	14,4	15,4	11,4	13,6	14,5
Doença renal crônica (%)	4,6	2,0	4,3	2,6	3,4	3,0
Insuficiência cardíaca congestiva (%)	20,5	18,3	21,3	19,0	19,8	21,1
**Região**						
Sudeste	86,8	85,9	85,6	84,3	85,7	85,9
Central	6,9	8,2	8,5	9,8	8,4	8,6
Sul e Nordeste	6,3	6,0	5,9	5,9	6,0	5,5
**Medicamentos concomitantes**						
Fenprocoumona	11	6	5	11	33	26
Aspirina	10	6	15	0	31	25
Clopidogrel	7	5	2	2	16	9
Aspirina + clopidogrel	3	0	1	0	4	2
Estatinas	27	29	33	29	118	99
Nitrato	2	3	5	6	16	14
Amiodarona	1	3	3	3	10	7
**Seguimento**						
Meses de monitorização (média/±DP)	13,87 (± 7,37)	16,04 (± 7,29)	18,02 (± 7,02)	17,24 (± 7,11)	16,30 (± 7,36)	18,13 (± 6,45)
Meses de monitorização (mediana/IIQ)	14,00 (7,0-20,0)	17,00 (9,0-23,0)	21,00 (12,0-24,0)	19,00 (11,0-23,0)	18,00 (10,0-23,0)	20,00 (13,0-23,0)

CHA_2_DS_2_-VASc: insuficiência cardíaca congestiva, hipertensão, idade, diabetes mellitus, acidente vascular cerebral/AIT, doença vascular, idade, categoria sexual; IIQ: Intervalo interquartil; DP: desvio padrão; TTR: tempo na faixa terapêutica.

### Controle de anticoagulação

**Figura 2:**
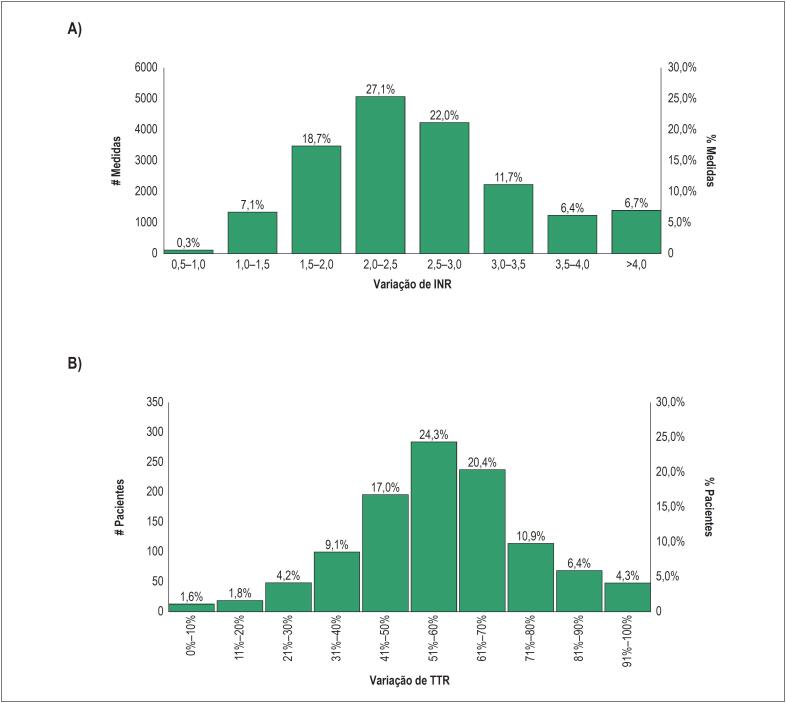
Resultados de INR e TTR. A. Distribuição das medidas por faixa de INR. B. Distribuição de pacientes por variação de TTR.

Cada paciente teve uma média de 15,63 (± 9,13) testes de RNI durante uma mediana de 18 meses de seguimento, equivalente a aproximadamente 0,95 testes por mês. O valor médio da RNI foi de 2,60 ± 0,88, o valor mediano da RNI foi de 2,44 (IIQ: 1,99 – 3,00). Entre todos os valores de RNI medidos, 49,1% estavam dentro da faixa terapêutica (2,0-3,0), enquanto 26,1% de todos os valores de RNI foram < 2,0 e 24,8% > 3,0 (Figura 2A). A mediana e a média dos níveis de TTR dos pacientes foram 58% (IIQ 47% -68%) e 56,6% (± 18,9%), respectivamente. A distribuição de TTR é mostrada na Figura 2B. Apenas 377 pacientes (31%) exibiram controle bom (TTR ≥ 65%), e 843 pacientes (69%) apresentaram controle abaixo do ideal (TTR < 65%).

### Desfechos clínicos

**Figura 3:**
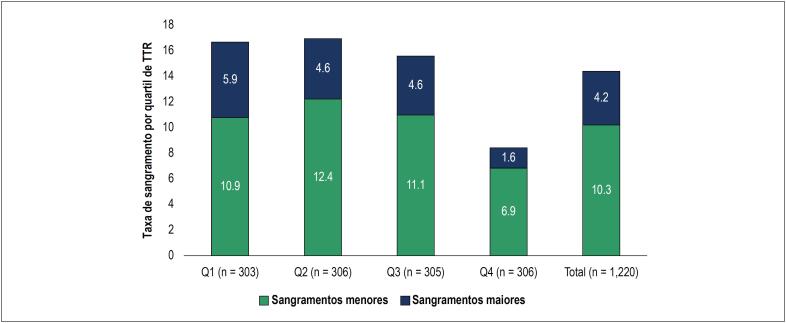
Taxa de sangramento por quartil de TTR.

Entre todos os pacientes, as taxas de sangramentos maiores e menores dos pacientes no programa foram de 4,2% e 10,3%, respectivamente (Figura 3). A taxa de sangramento maior entre pacientes bem controlados (TTR ≥ 65) foi de 1,6%, enquanto para pacientes com controle abaixo do ideal (TTR < 65%) foi de 5,3%. Portanto, a taxa de sangramento maior foi 3,3 vezes maior nos pacientes com controle abaixo do ideal quando comparados aos pacientes com controle bom (p < 0,01). Embora a tendência não tenha sido tão forte com sangramentos menores, um menor número de sangramentos menores foi observado nos subgrupos com TTR maior.

Uma análise exploratória foi realizada para observar o valor mais próximo da RNI antes do evento em uma amostra de pacientes admitidos devido a um acidente vascular cerebral. Dos 15 pacientes, 12 (80%) apresentaram acidente vascular cerebral hemorrágico ou inespecífico, apesar de apresentarem um RNI dentro da faixa terapêutica 2-3 (Informações suplementares).

### Resultados econômicos

O custo PMPY em toda a coorte foi de R$32.284 (US$10.679). Os custos de internação representaram aproximadamente 64% de todos os custos (R$20.710 ou US$6.851); os custos ambulatoriais representaram aproximadamente 36% (R$11.573 ou US$3.828). O custo médio de monitoramento da RNI PMPY foi de R$362 (US$120), variando de R$296 (US$98) a R$417 (US$138) e representando < 1% dos custos diretos totais (Tabela 2).

**Tabela 2:** Custos de PMPY com e sem grandes sangramentos (R $)

Valores. *Os custos são expressos como (média/±DP)*	Q1	Q2	Q3	Q4	Total
**Todos os pacientes**					
Número de pacientes	303	306	305	306	1220
Custo por paciente (total)	39,171 (± 59,728)	33,996 (± 48,637)	31,797 (± 42,030)	24,236 (± 35,158)	32,284 (± 47,480)
Custo por paciente ambulatorial	14,417 (± 31,295)	11,425 (± 18,544)	11,760 (± 17,866)	8,719 (± 12,084)	11,573 (± 21,218)
Custo por paciente internado	24,754 (± 45,652)	22,570 (± 43,267)	20,037 (± 40,199)	15,517 (± 35,849)	20,710 (± 41,725)
Custo de INR por paciente	296 (± 187)	405 (± 223)	417 (± 214)	329 (± 194)	362 (± 211)
**Sem sangramentos maiores**					
Número de pacientes	285	292	291	301	1169
Custo por paciente (total)	36,704 (± 58,663)	33,217 (± 49,138)	30,244 (± 40,852)	24,106 (± 35,376)	30,981 (± 46,858)
Custo por paciente ambulatorial	13,957 (± 31,658)	11,381 (± 18,955)	11,771 (± 18,261)	8,672 (± 12,181)	11,409 (± 21,419)
Custo por paciente internado	22,747 (± 44,912)	21,835 (± 44,213)	18,473 (± 38,417)	15,434 (± 36,298)	19,572 (± 41,328)
Custo de INR por paciente	291 (± 182)	400 (± 220)	416 (± 216)	329 (± 195)	359 (± 210)
**Com sangramentos maiores**					
Número de pacientes	18	14	14	5	51
Custo por paciente (total)	78,236 (± 64,550)	50,248 (± 33,950)	64,092 (± 53,895)	32,072 (± 17,703)	62,145 (± 52,163)
Custo por paciente ambulatorial	21,698 (± 25,175)	12,343 (± 6,755)	11,540 (± 6,290)	11,565 (± 3,997)	15,348 (± 16,170)
Custo por paciente internado	56,538 (± 49,698)	37,905 (± 30,108)	52,552 (± 50,731)	20,507 (± 15,797)	46,796 (± 44,386)
Custo de INR por paciente	382 (± 247)	523 (± 259)	432 (± 190)	357 (± 164)	432 (± 231)

Fator de conversão: 0,33 USD/BRL

O custo PMPY foi de R$25.352 (± R$37.762) ou US$8.386 (± US$12.492) por paciente bem controlado (TTR ≥ 65%) e R$35.384 (± R$50.900) ou US$11.705 (± US$16.838) por paciente com controle abaixo do ideal (TTR < 65%). Assim, pacientes com controle sub-ótimo de varfarina foram associados a custos 40% maiores, em média (p < 0,01).

Os custos PMPY com e sem sangramentos maiores foram de R$62.145 (US$20.558) e R$30.981 (US$10.249), respectivamente. Em todos os casos, os custos hospitalares foram maiores que os custos ambulatoriais (Tabela 2).

### Métricas por quartil de TTR

Algumas características e desfechos importantes foram observados nos quartis de TTR para verificar quais, caso existissem, eram mais prevalentes em pacientes com menor TTR em comparação com a população geral e pacientes com maior TTR. Conforme mostrado na Tabela 1, os pacientes com TTR mais baixo eram com mais frequência mulheres, apresentaram mais comorbidades (diabetes, doença renal, insuficiência cardíaca), menos testes de RNI e um período de monitoramento geral mais baixo.

### Análises de sensibilidade

Um total de 934 pacientes foram incluídos nas análises de sensibilidade. Um valor médio idêntico de RNI de 2,60 ± 0,96 e um valor mediano semelhante de RNI (2,43; IIQ: 2,00-3,00) foram observados nesse grupo de pacientes. As medianas e médias dos níveis de TTR foram quase as mesmas, 57% (IIQ 45% -68%) e 58% ± 16,2%, respectivamente. Nesse grupo de pacientes, os custos PMPY, incluindo pacientes internados e ambulatoriais, também foram bastante semelhantes, R$31.229 (US$10.331), *versus* R$32.284 (US$10.680) para a análise principal.

## Discussão

No geral, foi observado que a qualidade do controle da anticoagulação estava abaixo do ideal: apenas metade de todos os valores de RNI obtidos estavam na faixa terapêutica (RNI: 2-3), e os pacientes passavam um pouco mais da metade do tempo dentro da faixa terapêutica. O TTR variou em toda a população, e até dois terços dos pacientes não estavam adequadamente controlados (TTR < 65%). Esses pacientes foram associados a resultados clínicos e econômicos mais desfavoráveis, ou seja, mais sangramentos maiores e custos mais altos.

Dados epidemiológicos sugerem que houve mais de 700.000 AVCs no Brasil em 2010, representando mais de 141.000 mortes.^18^ Embora existam várias causas subjacentes ao AVC, estima-se que aproximadamente 20% dos acidentes vasculares cerebrais isquêmicos sejam atribuíveis à fibrilação atrial,^19^ e AVCs com fibrilação atrial tendem a ser maiores e associados a piores desfechos.^20^

A terapia de anticoagulação tem o potencial de reduzir bastante o risco de derrame em pacientes com fibrilação atrial. Foi demonstrado que a varfarina reduz o risco de acidente vascular cerebral isquêmico em 64% e a mortalidade em 26%, mas a utilidade da varfarina é variável devido à estreita faixa terapêutica, com o risco de eventos isquêmicos aumentando quando a RNI está abaixo de 2 e o risco de eventos hemorrágicos acima de 3.5.^21^

Os custos associados aos acidentes vasculares cerebrais são significativos e sustentados. Estimou-se que o custo de acidentes vasculares cerebrais isquêmicos em 2008 no Brasil foi de US$329 milhões, o custo de internação por paciente foi de US$1902 e o tempo médio de permanência foi superior a 13 dias.^20^ Os eventos hemorrágicos também representam um custo substancial como parte do manejo global do risco de AVC em pacientes com fibrilação atrial que recebem tratamento com anticoagulante oral.^22^ Um estudo nos EUA mostrou que a não adesão e a subutilização de varfarina pelos pacientes segurados com FA têm um impacto negativo na saúde e nos custos. Também foi demonstrado que o grau de controle da anticoagulação está diretamente correlacionado a melhores resultados para pacientes com fibrilação atrial em tratamento com varfarina.^23^^-^^25^

Poucos estudos avaliaram a extensão do controle da anticoagulação com varfarina em países da América Latina. Pesquisas anteriores relataram níveis próximos do aceitável do controle de anticoagulação no Brasil, com níveis de TTR próximos a 60% em ambientes controlados^26^^-^^28^ e entre 60 e 65% no mundo real.^9^^-^^11^ No entanto, esses estudos foram realizados principalmente em um ou dois hospitais públicos ou clínicas de anticoagulação, em populações com tamanho limitado de amostra e amplo uso de varfarina.

O TTR é a medida aceita do controle da anticoagulação em pacientes com varfarina, e está correlacionado com os desfechos clínicos. Embora seja frequentemente relatado por centro ou mesmo por país em ensaios clínicos, há uma heterogeneidade substancial no TTR de cada paciente.^29^^,^^30^ Os resultados do presente estudo são consistentes com esse conceito, pois mesmo com a população geral de pacientes apresentando um TTR razoável, a maioria dos pacientes apresentou, na realidade, um TTR abaixo do limiar considerado ideal.^23^

O presente estudo promove a compreensão do modelo de assistência em anticoagulação na prática clínica de rotina. É representativo de uma população relativamente jovem com FA, apresentando uma prevalência mais baixa de comorbidades do que a relatada em outros estudos observacionais e ambientes controlados.^26^^-^^28^^,^^31^ Além disso, o estudo é representativo de dados do mundo real em um ambiente hospitalar específico da AMIL, incluindo um programa estruturado e chamadas telefônicas, e não é generalizável para outros ambientes hospitalares, como o setor público. A abordagem para gerenciar e monitorar regularmente os pacientes através do programa de atendimento foi considerada bastante singular. Estudos que abordaram uma pergunta de pesquisa semelhante^9^^-^^11^ não relataram a existência de um programa tão dedicado para os pacientes tratados com varfarina. A monitorização da RNI foi realizada aproximadamente uma vez por mês, uma frequência maior do que em outros estudos observacionais^32^, embora menor do que em ambientes controlados.^26^ Apesar do seguimento regular, apenas cerca de metade (49,1%) de todos os valores de RNI obtidos estavam na faixa terapêutica, e uma parcela limitada da população apresentou um controle bom de TTR. Os resultados da TTR foram consistentes com pesquisas anteriores na prática assistencial, indicando que os pacientes recebendo varfarina passam apenas um pouco mais da metade do tempo dentro da faixa terapêutica.^32^ Os níveis de TTR relatados para a população geral tratada com varfarina neste estudo estavam ligeiramente abaixo do limite inferior da faixa recomendada.^9^^,^^11^^,^^25^ Um achado interessante é que a distribuição de TTR na Figura 2B mostrou uma inclinação para a direita, o que significa que havia um nicho de pacientes com controle muito alto do TTR. Cerca de 22% dos pacientes apresentaram TTR > 70%.

Dados internacionais que avaliaram a associação entre controle da anticoagulação e desfechos do uso de varfarina indicam que pacientes com controle deficiente de varfarina apresentam resultados clínicos e econômicos mais desfavoráveis do que pacientes bem controlados.^21^^,^^33^ Os resultados do presente estudo estão bastante alinhados com trabalhos anteriores e contribuem ainda mais para o entendimento de como o controle da varfarina pode impactar tanto nos eventos clínicos quanto nos custos na prática brasileira de rotina. A alta qualidade do controle da anticoagulação foi associada a uma menor incidência de sangramentos maiores e menores e a uma economia substancial dos custos médicos diretos, devido à redução dos custos hospitalares e ambulatoriais. Pacientes com controle abaixo do ideal apresentaram 3,3 vezes mais sangramentos maiores e custos PMPY 40% mais altos do que pacientes bem controlados.

Apesar do tratamento com anticoagulação, os acidentes vasculares cerebrais ainda ocorrerão como foram observados neste estudo, tanto isquêmicos quanto hemorrágicos. É importante ressaltar que, dentre 10 derrames hemorrágicos confirmados identificados neste estudo, o valor de RNI prévio para 7 dos 10 eventos estava dentro da faixa terapêutica de 2 a 3, sendo que os outros 3 apresentaram valores de 3,66, 3,87 e 5,13. Isso é consistente com os achados de uma subanálise do estudo ARISTOTLE, que mostrou que para cerca de 80% das hemorragias intracranianas que ocorreram em pacientes tratados com varfarina, o RNI prévio estava entre 2 e 3.^34^

Uma pesquisa anterior explorou preditores de TTR baixo,^6^^,^^26^^,^^32^ sugerindo que pacientes com menor TTR na maioria das vezes eram mulheres, tinham menos escolaridade e mais comorbidades, especificamente diabetes, doença renal crônica, insuficiência cardíaca e acidente vascular cerebral anterior. Com bastante consistência neste estudo, pacientes do sexo feminino e pacientes com mais comorbidades, como doença renal crônica e cardiopatia isquêmica, também tenderam a apresentar valores mais baixos de TTR. Além disso, pacientes com menor TTR tinham menos testes de RNI e um período de monitoramento global mais curto. Os resultados sugerem que é necessário identificar pacientes com labilidade das RNIs e avaliar mais oportunidades para melhorar seu TTR, como educação ou seguimento mais próximo. Caso contrário, outras formas de anticoagulação, como a classe de anticoagulante não-vitamina K, aprovada mais recentemente, devem ser consideradas. Essa classe não requer monitoramento de rotina, possui menos interações medicamento-medicamento e medicamento-alimento do que a varfarina e demonstrou ser pelo menos tão segura e eficaz quanto a varfarina bem controlada, além de apresentar menor taxa de hemorragia intracraniana.^35^

### Limitações

Nosso estudo tem vários pontos fortes e limitações. A coorte de pacientes do estudo foi uma das maiores até agora entre os estudos do mundo real no Brasil. O uso combinado de solicitações e o programa de assistência agregaram valor significativo ao estudo, principalmente ao permitir a análise dos valores de RNI, geralmente não disponíveis nas solicitações. No entanto, dada a sua natureza observacional retrospectiva, apenas associações puderam ser concluídas. Este estudo observou variações do TTR ao longo do tempo e, por isso, foi vulnerável aos efeitos de medições repetidas como uma intervenção. Nenhuma técnica estatística avançada foi utilizada para equilibrar as características dos subgrupos de TTR nos pacientes e, portanto, nenhuma conclusão inferencial sobre cofatores pôde ser obtida. Não foi possível calcular o escore médio de risco HAS-BLED, pois nem todos os pontos de dados dos componentes do escore foram capturados no conjunto de dados (por ex., etilismo). A incidência de outros desfechos como acidente vascular cerebral, mortalidade, descontinuação e aderência não foi analisada. Não foram realizadas análises de sensibilidade em outros limiares específicos de TTR (por exemplo, 60% ou 70%). A estabilidade da RNI ao longo do tempo não foi avaliada. Somente custos médicos diretos estavam disponíveis; estes se referiam aos custos de todas as causas incorridos por cada paciente, desconsiderando o motivo da utilização e, consequentemente, podem ter sido superestimados. A utilização de recursos de saúde e subgrupos de pacientes não foram avaliados.

De acordo com as normas brasileiras para códigos de procedimento (Apêndice A), a RNI não possui código individual, mas está incluída no código “Teste de coagulação”. Como não foi possível segregar, a medida da RNI foi considerada como teste de coagulação completo, e não como uma porcentagem, para todos os pacientes.

Verificou-se que uma parcela significativa dos pacientes em uso de varfarina (11%) apresentou escore CHA2DS2-VASC igual a zero, superior à porcentagem relatada em outros estudos (6,1%).36 A avaliação do CHA2DS2-VASC está sujeita à documentação clínica do histórico dos pacientes, considerando que detalhes das condições pré-existentes podem ter sido subnotificados.

O programa de monitoramento por telefone foi oferecido a pacientes de uma companhia de seguros de saúde específica e, quando o contrato de um paciente foi rescindido, o acompanhamento não foi possível.

Finalmente, algumas das limitações do estudo são inerentes a um desenho de estudo observacional retrospectivo. Isso inclui possíveis erros de codificação e dados ausentes, os quais podem ter introduzido vieses no estudo e afetado o número de pacientes excluídos, e o fato de os dados avaliados não terem sido originalmente coletados para fins de pesquisa clínica.

## Conclusões

Este estudo examinou o perfil dos pacientes, a qualidade da anticoagulação e os desfechos clínicos/econômicos em pacientes tratados com varfarina para FANV em uma companhia privada de seguros de saúde no Brasil. É representativo de uma coorte grande e relativamente jovem de pacientes tratados com varfarina. A qualidade geral do manejo da anti-coagulação ficou abaixo do ideal. Os pacientes recebendo varfarina ficaram dentro da faixa terapêutica por pouco mais da metade do tempo. Até dois terços apresentaram controle abaixo do ideal (TTR < 65%) e isto estava associado a mais eventos de sangramento e custos maiores. Essa análise destaca a importância, em termos de resultados e custos, de um rígido controle de anticoagulação para pacientes com FANV tratados com varfarina, e a dificuldade em manter um TTR adequado, mesmo com um programa bem projetado e executado. Pesquisas adicionais são necessárias, à medida que mais dados do mundo real se tornam disponíveis, para avaliar mais profundamente o uso de varfarina, bem como a adoção de Novos Anticoagulantes Orais (NOACS) *versus* varfarina.
