# Supplementary material for: Terapia de Anticoagulação em Pacientes com Fibrilação Atrial não Valvar em Ambiente de Cuidado de Saúde Privado no Brasil: Um Estudo de Mundo Real
Source: Arq Bras Cardiol. 2020 Apr 6;114(3):457–66. [Article in Portuguese] doi: 10.36660/abc.20180076 (PMC7792730; doi:10.36660/abc.20180076)
Supplement: Translation

# Translation: Anticoagulation Therapy in Patients with Non-valvular Atrial Fibrillation in a Private Setting in Brazil: A Real-World Study

**Authors:** Pedro Gabriel Melo de Barros e Silva, Henry Sznejder, Rafael Vasconcellos, Georgette M. Charles, Hugo Tannus F. Mendonca-Filho, Jack Mardekian, Rodrigo Nascimento, Stephen Dukacz, Manuela Di Fusco

**Affiliations:** 1 São PauloSP Brazil Hospital Samaritano Paulista, São Paulo, SP – Brazil; 2 Rio de JaneiroRJ Brazil United Health Group-Brasil, Rio de Janeiro, RJ – Brazil; 3 MinnetonkaMinnesota USA United Health Group, Minnetonka, Minnesota – USA; 4 Eden PrairieMinnesota USA Optum Life Sciences, Eden Prairie, Minnesota – USA; 5 New York USA Pfizer Inc., New York – USA; 6 São PauloSP Brazil Pfizer Inc., São Paulo, SP – Brazil

**Keywords:** Warfarine/therapeutic use, Anticoagulants/adverse effects, Atrial Fibrillation/comlications, Hospitals, Private/ economics, Health Care Quality, Access and Evaluation

## Abstract

**Background:**

The safety and effectiveness of warfarin depend on anticoagulation control quality. Observational studies associate poor control with increased morbidity, mortality and healthcare costs.

**Objectives:**

To develop a profile of non-valvular atrial fibrillation (NVAF) patients treated with warfarin in a Brazilian private ambulatory and hospital setting, evaluate the quality of anticoagulation control, and its association with clinical and economic outcomes.

**Methods:**

This retrospective study, through a private health insurance dataset in Brazil, identified NVAF patients treated with warfarin between 01 MAY 2014 to 30 APRIL 2016, described their anticoagulation management, and quantified disease-related costs. Data on demographics, clinical history, concomitant medication and time in therapeutic range (TTR) of international normalized ratio (INR) values were retrieved. Patients were grouped into TTR quartiles, with good control defined as TTR ≥ 65% (Rosendaal method). Major bleeds and all-cause direct medical costs were calculated and compared between good and poor control subgroups. P-values < 0.05 were considered statistically significant.

**Results:**

The analysis included 1220 patients (median follow-up: 1.5 years; IQR: 0.5–2.0). On average, each patient received 0.95 monthly INR measurements (mean INR: 2.60 ± 0.88, with 26.1% of values < 2 and 24.8% > 3), (median TTR: 58%; IQR: 47–68%), (mean TTR: 56.6% ± 18.9%). Only 31% of patients were well-controlled (mean TTR: 78% ± 10%), with 1.6% having major bleeds within median follow-up, and direct medical costs per member per year (PMPY) of R$25,352(± R$ 37,762). Poorly controlled patients (69%) were associated with 3.3 times more major bleeds (5.3% vs. 1.6%; p < 0.01) and 40% higher costs (R$35,384 vs. R$25,352; p < 0.01).

**Conclusions:**

More than 60% of the patients were below the desired target and the associated costs were higher.

## Introduction

Atrial fibrillation (AF) is the most common sustained cardiac arrhythmia that affects more than 33 million people worldwide. Most cases are non-valvular AF (NVAF) patients.^1^^-^^3^ Epidemiology data for AF in Latin America is limited and a significant proportion of patients has poor control of key risk factors and does not receive appropriate anticoagulation treatment (18.3% – 24.6%).^4^^,^^5^

Clinical guidelines recommend the use of an oral anticoagulant (OAC) in NVAF to reduce the risk of stroke.^2^^,^^3^ For decades, vitamin K antagonists (VKAs), the most commonly used of which is warfarin, have been the cornerstone of OAC therapy for NVAF. However, the safety and efficacy of warfarin have limitations and depend on the tight quality of anticoagulation control.^2^ This is achieved using a standardized measure of clotting time known as the international normalized ratio (INR), which is desired to be between 2 and 3.^6^ Frequent INR monitoring and dose adjustment are needed to maintain target INR levels.^2^^,^^3^ However, monitoring can increase the medical and economic burden.^7^

Time in therapeutic range (TTR) is the standard means of assessing the long-term quality of anticoagulation control and the risk–benefit profile of warfarin.^6^ TTR represents the proportion of time that a patient's INR values are between 2 and 3, having the maximum benefit when the TTR is 60% to 70% or higher.^2^ In Latin America, the median TTR was at the lower end of the recommended levels of anticoagulation (near 60%).^4^^,^^8^ In Brazil, some observational studies in the public setting showed that most patients had good anticoagulation control, though TTR levels were in the lower end of the threshold.^9^^-^^11^ Managing OAC use, including INR monitoring, are costly and inaccessible for many patients in Latin America.^4^ To date, few studies have been conducted in private settings in the region. Associations of TTR levels with clinical or economic outcomes were generally not reported. The objective of this study was to develop a profile of patients receiving warfarin for NVAF in a private setting in Brazil, and to evaluate the quality of anticoagulation control and clinical/economic outcomes.

## Methods

### Data Sources

Data from May 1, 2014 to April 30, 2016 were pulled from a large private health insurance dataset in Brazil – AMIL. AMIL is one of the largest health insurance companies in Brazil, with over 4 million beneficiaries and clinical care programs with integrated and structured information of prevalent diseases. The AMIL dataset combines electronic medical records containing information on patient demographics, enrolment and clinical history, with medical claims from outpatient and inpatient hospital admissions, ambulatory care facilities and emergency departments.

For warfarin-treated patients, AMIL runs a private anticoagulation phone monitoring program named VIVA AMIL.^12^ Within this program, trained nurses and nursing technicians make monthly phone calls to patients to collect patient data, self-reported results of the last INR test, occurrence of thromboembolic and bleeding events, medication regularity and adverse effects.

An existing template, created to capture data from the monitored patients, was used to ensure the test results, experienced events and patterns were reported consistently to meet the program needs. An initial call was made to collect clinical and demographic data (if otherwise not available), including the presence of chronic conditions and medications under use. Each patient then received monthly outbound calls, but patients also had the option to call as needed.

In case the patient did not have a current or recent INR test result, a nurse would support them by requesting the test and reminding them to call back and report the results. In the situation in which the INR results reported by the patient were out of the target range (INR 2-3), the nurse would discuss dose adjustments with the patient and advise them to seek medical advice in person.

### Patient Selection

Patients aged 18 or older were included if they had an AF diagnosis (ICD-10-CM code I48) or were assessed for AF in a specific system form in the electronic medical record, if they received at least one prescription for VKA during the study period, had continuous health plan coverage and if they were followed by the phone-monitoring program for at least 4 months with a record of the calls in at least 50% of the months during the study period. Patients with evidence of moderate/severe mitral stenosis, VTE or a mechanical prosthetic valve were excluded. The research protocol was approved by the local Institutional Review Board.

### Variables and Outcome Measures

Key characteristics of patients receiving warfarin were analyzed from claims, electronic medical records and self-reports: demographics and clinical history (CHA_2_DS_2_-VASc score, comorbidities, prior stroke or bleeds, INR and TTR). Specifically, patients were classified as having chronic renal failure when there was at least one of the selected ICD-10 codes (Appendix A) linked to them in the dataset during the entire study period, or if chronic renal failure was present in the data collection form managed by the nurse. Concomitant medication utilization and INR frequency patterns were also assessed.

Consistent with guidelines and prior studies,^2^^,^^6^ the quality of INR control was based on the percentage of time during which a patient receiving warfarin was within therapeutic range (2.0-3.0) over the entire follow-up period. Good control was defined as TTR ≥ 65%. The number of INR tests for each patient was obtained through the claims dataset, which did not record the INR values. During the phone monitoring calls, the trained nurse would ask the patient to report the values of the INR tests undertaken since the last call. The INR test frequency was used to calculate the total and mean INR tests per patient. Since the INR is a low-complexity and low-cost procedure, the test could have been paid out-of-pocket by the patient and therefore not reported in claims. In order to reduce the impact of unstated INR tests, during the phone monitoring calls the nurse would ask the patient to also report the date of the INR test, along with the INR values. For those cases in which a corresponding claim was absent, the nurse would manually add the test frequency information in the electronic medical record. TTR was calculated using the Rosendaal method, computed using the INR values that were recorded in the electronic medical records.^13^

The clinical outcomes assessed were major and minor bleeding events, identified using the ICD-10 codes of inpatient claims listed in Appendix A.^14^ Self-reported situations were also considered. The diagnosis codes used for major bleeds were based on a validated administrative claim-based algorithm, as well as the International Society on Thrombosis and Hemostasis definition of major bleeding.^15^^,^^16^ Bleeding rates were calculated as the number of patients with at least one self-reported bleeding episode during the monitoring period, divided by the total number of patients. To assess the outcomes, patients were followed until April 30, 2016, unless health plan disenrollment or death occurred first.

All-cause direct medical costs were assessed from the claims of each patient for office elective visits, emergency department visits, outpatient tests/procedures, inpatient admissions, and home health/care transition admissions. The costs represented the actual costs borne by the insurance provider (AMIL). Out-of-pocket costs were not included. The costs were available in the data source over the study period and were annualized by dividing them by the months of the study period and multiplying them by 12. After this calculation, costs were expressed per member per year (PMPY), in Brazilian Reals (R$) and converted to US dollars. A conversion factor of 0.33 USD/BRL was obtained by averaging the daily exchange rates within each year of the study period (May 1, 2014 to April 30, 2016). The daily exchange rates were obtained from historical records of a public currency exchange calculator.^17^

Out-of-pocket costs were not included. Finally, key characteristics, clinical and economic outcomes were observed and compared amongst TTR quartiles.

### Statistical methods

Due to the exploratory nature of the study, key characteristics and outcomes were descriptively analyzed.

Descriptive statistics were reported as counts, percentages, means, medians, standard deviations and quartiles. Continuous variables were described as mean and standard deviation or median and respective interquartile range, depending on whether or not a normal distribution was found. Categorical variables were described as frequencies and percentages. Comparisons were made between continuous variables using an independent unpaired two-sample *t*-test and between categorical variables using the chi-square test. P-values < 0.05 in two-tailed tests were considered statistically significant. All analyses were carried out using SAS 9.4.

### Subgroup analysis

The key characteristics, clinical and economic outcomes were analyzed for the overall population and for patients with poor (TTR < 65%) and good (TTR ≥ 65%) control.

### Sensitivity analysis

To check for main analysis consistency, some patient characteristics, INR, TTR levels and PMPY costs were observed for a group of patients followed for at least 6 months with records of the calls in at least 50% of the months during the study period.

## Results

### Patient characteristics

A total of 1,220 patients with NVAF were included for the main analysis (Figure 1). Overall, median follow-up was 1.5 years (interquartile range [IQR]: 0.5–2.0 years). Key patient characteristics are listed in Table 1. The mean age was 63.9 ± 14.7 years and 50.7% were females. The mean CHA_2_DS_2_-VASc score was 2.45 ± 0.88. Most patients (85.7%) were from the Southeast region of Brazil. Approximately 10% of patients were on concomitant statin therapy and a minority of patients (~4%) were receiving concomitant antiplatelet therapy with aspirin and/or Clopidogrel. Hypertension was the most prevalent comorbidity (38.5%), followed by heart failure (19.8%), prior stroke (13.7%) and diabetes (13.6%).

**Figure 1:** Flow chart describing inclusion and exclusion criteria.

**Table 1:** Patient characteristics and metrics per TTR quartile

Values	Period (months)					
			4 – 24			6 – 24 (Sensitivity analysis)
	Q1 N = 303	Q2 N = 306	Q3 N = 305	Q4 N = 306	Total N = 1220	N = 934
**Demographics**						
Age (mean/±SD)	62.02 (±15.92)	64.58 (±13.83)	64.49 (±14.48)	64.30 (±14.63)	63.85 (±14.75)	64.75 (±14.03)
Female (%)	50.8	55.2	52.5	44.1	50.7	51.5
**Anticoagulation**						
INR (mean/±SD)	2.56 (± 1.25)	2.67 (± 1.10)	2.61 (± 0,89)	2.54 (± 0,62)	2.60 (±0.88)	2.60 (±0.96)
INR (median/IQR)	2.22 (1.70-3.16)	2.50 (1.97-3.20)	2.48 (2.06-2.98)	2.44 (2.13-2,78)	2.44 (1.99-3.00)	2.43 (2.00-3.00)
TTR (mean/±SD)	32.6% (±11.5%)	51.2% (±3.3%)	62.0% (±3.2%)	80.2% (±9.8%)	56.6% (±18.9%)	58.0% (±16.2%)
TTR (median/IQR)	36% (28-42%)	52% (48-54%)	62% (59-65%)	78% (72-86%)	58% (47-68%)	57.0% (45-68%)
INR tests per patient (mean/±SD)	12.79 (±8.09)	17.49 (±9.65)	18.00 (±9.27)	14.20 (±8.40)	15.63 (±9.13)	18.44 (±8.60)
**Risk factors and baseline conditions**						
CHA_2_DS_2_-VASc (mean/±SD)	2.38 (±1.72)	2.46 (±1.69)	2.55 (±1.69)	2.44 (±1.74)	2.45 (±1.71)	2.58 (±1.72)
Stroke (%)	13.9	12.1	11.5	17.3	13.7	14.6
Hypertension (%)	33.3	39.2	43.6	37.9	38.5	41.3
Diabetes (%)	13.2	14.4	15.4	11.4	13.6	14.5
Chronic kidney failure (%)	4.6	2.0	4.3	2.6	3.4	3.0
Congestive heart failure (%)	20.5	18.3	21.3	19.0	19.8	21.1
**Region**						
Southeast	86.8	85.9	85.6	84.3	85.7	85.9
Central	6.9	8.2	8.5	9.8	8.4	8.6
South and Northeast	6.3	6.0	5.9	5.9	6.0	5.5
**Concomitant medications**						
Phenprocoumon	11	6	5	11	33	26
Aspirin	10	6	15	0	31	25
Clopidogrel	7	5	2	2	16	9
Aspirin + clopidogrel	3	0	1	0	4	2
Statins	27	29	33	29	118	99
Nitrate	2	3	5	6	16	14
Amiodarone	1	3	3	3	10	7
**Follow-up**						
Months of monitoring (mean/±SD)	13.87 (± 7.37)	16.04 (± 7.29)	18.02 (± 7.02)	17.24 (± 7.11)	16.30 (± 7.36)	18.13 (± 6.45)
Months of monitoring (median/IQR)	14.00 (7.0-20.0)	17.00 (9.0-23.0)	21.00 (12.0-24.0)	19.00 (11.0-23.0)	18.00 (10.0-23.0)	20.00 (13.0-23.0)

CHA_2_DS_2_-VASc: congestive heart failure, hypertension, age, diabetes mellitus, stroke/TIA, vascular disease, age, sex category; IQR: interquartile range; SD: standard deviation; TTR: time in therapeutic range.

### Anticoagulation control

Each patient had a mean of 15.63 (±9.13) INR tests over a median of 18 months of follow-up, equivalent to approximately 0.95 tests per month. The mean INR value was 2.60± 0.88, the median INR value was 2.44 (IQR: 1.99 – 3.00). Among all measured INR values, 49.1% were within the therapeutic range (2.0–3.0), whereas 26.1% of all INR values were < 2.0, and 24.8% were > 3.0 (Figure 2A). The median and mean patient-level TTRs were 58% (IQR 47%–68%) and 56.6% (±18.9%), respectively. The TTR distribution is reported in Figure 2B. Only 377 patients (31%) exhibited good control (TTR ≥ 65%) and 843 patients (69%) had poor control (TTR < 65%).

**Figure 2:** INR and TTR results. A. Measurement distribution per INR range. B. Patient distribution per TTR range.

### Clinical outcomes

Among all patients, the major and minor bleeding rates of patients in the program were 4.2% and 10.3%, respectively (Figure 3). The major bleeding rate among well-controlled patients (TTR ≥ 65) was 1.6%, whereas it was 5.3% for poorly controlled patients (TTR < 65%). Therefore, the major bleeding rate was 3.3 times higher in poorly-controlled patients when compared with well-controlled patients (p < 0.01). While the trend was not as strong with minor bleedings, fewer minor bleeds were observed in subgroups with highest TTR.

**Figure 3:** Bleeding rate per TTR quartile.

An exploratory analysis was conducted to observe the closest INR value prior to the event on a sample of patients admitted for a stroke. Out of 15 patients, 12 (80%) experienced a hemorrhagic or unspecified stroke event, despite having an INR within the therapeutic range 2-3 (Supplementary information).

### Economic outcomes

The PMPY cost across the entire cohort was R$32,284 (USD$10,679). Inpatient costs represented ~64% of all costs (R$20,710 or USD$6,851); outpatient costs represented ~36% (R$11,573 or USD$3,828). The mean INR monitoring cost PMPY was R$362 (USD$120), ranging from R$296 (USD$98) to R$417 (USD$138) and representing < 1% of the total direct costs (Table 2).

**Table 2:** PMPY costs with and without major bleeds (R$)

Values. *Costs are expressed as (mean/±SD)*	Q1	Q2	Q3	Q4	Total
**All patients**					
Number of patients	303	306	305	306	1220
Cost per patient (total)	39,171 (± 59,728)	33,996 (± 48,637)	31,797 (± 42,030)	24,236 (± 35,158)	32,284 (± 47,480)
Cost per outpatient	14,417 (± 31,295)	11,425 (± 18,544)	11,760 (± 17,866)	8,719 (± 12,084)	11,573 (± 21,218)
Cost per inpatient	24,754 (± 45,652)	22,570 (± 43,267)	20,037 (± 40,199)	15,517 (± 35,849)	20,710 (± 41,725)
NR cost per patient	296 (± 187)	405 (± 223)	417 (± 214)	329 (± 194)	362 (± 211)
**Without major bleedings**					
Number of patients	285	292	291	301	1169
Cost per patient (total)	36,704 (± 58,663)	33,217 (± 49,138)	30,244 (± 40,852)	24,106 (± 35,376)	30,981 (± 46,858)
Cost per outpatient	13,957 (± 31,658)	11,381 (± 18,955)	11,771 (± 18,261)	8,672 (± 12,181)	11,409 (± 21,419)
Cost per inpatient	22,747 (± 44,912)	21,835 (± 44,213)	18,473 (± 38,417)	15,434 (± 36,298)	19,572 (± 41,328)
NR cost per patient	291 (± 182)	400 (± 220)	416 (± 216)	329 (± 195)	359 (± 210)
**With major bleedings**					
Number of patients	18	14	14	5	51
Cost per patient (total)	78,236 (± 64,550)	50,248 (± 33,950)	64,092 (± 53,895)	32,072 (± 17,703)	62,145 (± 52,163)
Cost per outpatient	21,698 (± 25,175)	12,343 (± 6,755)	11,540 (± 6,290)	11,565 (± 3,997)	15,348 (± 16,170)
Cost per inpatient	56,538 (± 49,698)	37,905 (± 30,108)	52,552 (± 50,731)	20,507 (± 15,797)	46,796 (± 44,386)
NR cost per patient	382 (± 247)	523 (± 259)	432 (± 190)	357 (± 164)	432 (± 231)

Conversion factor: 0.33 USD/BRL.

The PMPY cost was R$25,352 (± R$37,762) or USD$8,386 (± USD$12,492)per well-controlled patient (TTR ≥ 65%) and R$35,384 (± R$50,900) or USD$11,705 (± USD$16,838) per poorly-controlled patient (TTR < 65%). Thus, patients with suboptimal warfarin control were associated with 40% higher costs, on average (p < 0.01).

PMPY costs with and without major bleeds were R$62,145 (USD$20,558) and R$30,981 (USD$10,249), respectively. In all cases, inpatient costs were greater than outpatient costs (Table 2).

### Metrics per TTR quartile

Some key characteristics and outcomes were observed across TTR quartiles to see which, if any, were more prevalent in patients with lower TTR compared with the overall population and patients with higher TTR. As shown in Table 1, patients with lower TTR were more often females, had more comorbidities (diabetes, renal disease, heart failure), fewer INR tests and a lower overall monitoring period.

### Sensitivity analyses

A total of 934 patients were included in the sensitivity analyses. An identical mean INR value of 2.60 ± 0.96 and a similar median INR (2.43; IQR: 2.00-3.00) were observed in this patient group. The median and mean patient-level TTRs were almost the same, 57% (IQR 45%–68%) and 58% ± 16.2%, respectively. In this group of patients, PMPY costs, including inpatient and outpatient, were also quite similar, R$31,229 (USD$10,331), versus R$32,284 (USD$10,680) for the main analysis.

## Discussion

Overall, it was observed that the quality of anticoagulation management was suboptimal: only half of all INR values drawn were in the therapeutic range (INR: 2-3) and patients spent a bit more than half of the time within the therapeutic range. TTR varied across the population and up to two thirds of patients were not adequately controlled (TTR < 65%). These patients were associated with more unfavorable clinical and economic outcomes i.e. more major bleeds and higher costs.

Epidemiological data suggest that there were over 700,000 strokes in Brazil in 2010, accounting for over 141,000 deaths.^18^ While there are several underlying causes of stroke, it is estimated that approximately 20% of ischemic strokes are attributable to atrial fibrillation,^19^ and strokes associated with atrial fibrillation tend to be larger and associated with worse outcomes.^20^

Anticoagulation therapy has the potential to greatly reduce the risk of stroke in patients with atrial fibrillation. Warfarin has been shown to reduce the risk of ischemic stroke by 64% and mortality by 26% but the usefulness of warfarin is variable due to the narrow therapeutic range, with the risk of ischemic events increasing when the INR is below 2, and the risk of hemorrhagic events increasing above 3.5.^21^

Costs associated with strokes are significant and sustained. It was estimated that the 2008 cost of ischemic strokes in Brazil was $329 million USD, the per-patient cost of hospitalization was $1902 USD, and the mean length of stay was over 13 days.^20^ Hemorrhagic events also represent a substantial cost as part of the overall management of stroke risk for atrial fibrillation patients receiving oral anticoagulation treatment.^22^ A US study has shown that non-adherence and underuse of warfarin by insured patients with AF has a negative impact on health and costs. It has also been demonstrated that the degree of anticoagulation control is directly correlated to improved outcomes for patients with atrial fibrillation receiving warfarin treatment.^23^^-^^25^

Few studies have assessed the extent of anticoagulation control with warfarin in Latin American countries. Past research reported close to acceptable levels of anticoagulation control in Brazil, with TTR levels close to 60% in controlled settings^26^^-^^28^ and between 60 and 65% in the real world.^9^^-^^11^However, these studies were conducted mostly in one or two public hospitals or anticoagulation clinics, in populations with limited sample size and broad use of warfarin.

The TTR is the accepted measure of anticoagulation control for warfarin patients and is correlated with clinical outcomes. While often reported by center or even country in clinical trials, there is substantial heterogeneity in individual patient ^29^^,^^30^ The results from this current study are consistent with this concept in that even though the overall patient population had a fair TTR, in fact most of the patients had a TTR which was below the threshold considered optimal.^23^

The present study furthers the understanding of the anticoagulation care model in routine clinical practice. It is representative of a relatively young AF population presenting with a lower prevalence of comorbidities than what has been reported in other observational studies and controlled settings.^26^^-^^28^^,^^31^ In addition, the study is representative of real-world data in a specific private setting of AMIL, including a structured program and phone calls, and it is not generalizable to other settings like the public sector. The approach to managing and regularly monitoring the patients through the care program was found to be quite unique. Studies that addressed a similar research question^9^^-^^11^ did not report the existence of such a dedicated program for warfarin patients. INR monitoring was performed approximately once a month, more frequently than in other observational studies^32^ but less than in controlled settings.^26^ Despite the regular follow-up, only about half (49.1%) of all INR values drawn were in the therapeutic range and a limited portion of the population had good TTR control. The TTR results were consistent with past research within the care practice, indicating that warfarin patients spend only a bit more than half of the time within the therapeutic range.^32^ The reported TTR levels for the overall population treated with warfarin in this study were slightly below the lower limit of the recommended threshold interval.^9^^,^^11^^,^^25^ An interesting finding is that the TTR distribution in Figure 2B was skewed to the right, meaning that there was a niche of patients with very high TTR control. Around 22% of patients had TTR > 70%.

International data that assessed the association between warfarin control and outcomes indicate that poor warfarin control patients experience more unfavorable clinical and economic outcomes than well controlled patients.^21^^,^^33^ The results of the present study are quite aligned with prior work and further contribute to the understanding of how warfarin control could impact on both clinical events and costs in the Brazilian routine practice. High quality of anticoagulation control was associated with a lower incidence of major and minor bleeds and substantial direct medical cost savings from both reduced inpatient and outpatient costs. Poorly-controlled patients had 3.3 times more major bleeds and 40% higher PMPY costs than well-controlled patients.

Despite anticoagulation treatment, strokes will still occur, as observed in this study, both ischemic and hemorrhagic ones. Of note, out of 10 confirmed hemorrhagic strokes that were identified in this study, the preceding INR value for 7 of the 10 was within the therapeutic range of 2 to 3, with the other 3 being 3.66, 3.87, and 5.13. This is consistent with the findings from a sub-analysis of the ARISTOTLE trial which showed that for about 80% of the intracranial hemorrhages that occurred in warfarin-treated patients, the preceding INR was between 2 and 3.^34^

Past research explored predictors of poor TTR^6^^,^^26^^,^^32^ suggesting that patients with lower TTR were more often females, had less schooling and more comorbidities, specifically diabetes, chronic kidney disease, heart failure and prior stroke. Quite consistently, female patients and patients with more comorbidities such as chronic kidney disease and ischemic heart disease tended to have lower TTR values in this study, too. Moreover, patients with lower TTR had fewer INR tests and a shorter overall monitoring period. The results suggest that there is a need to identify patients with labile INRs and further assess opportunities to improve their TTR, such as education or closer follow-up. Failing that, other forms of anticoagulation such as the more recently approved non-vitamin K anticoagulant class should be considered. This class does not require routine monitoring, has fewer drug-drug and drug-food interactions than warfarin, and has been shown to be at least as safe and efficacious as well-controlled warfarin, and to have a lower rate of intracranial haemorrhage.^35^

### Limitations

Our study has several strengths and limitations. The patient cohort of the study was one of the largest thus far among real-world studies in Brazil. The combined use of claims and the care program added significant value to the study, especially by allowing the analysis of INR values, commonly not available in claims. However, given its retrospective observational nature, only associations could be concluded. This study observed TTR variations over time and as such was vulnerable to the effects of repeated measurements as an intervention. No advanced statistical techniques were used to balance characteristics of the TTR patient subgroups and therefore no inferential conclusions about cofactors could be drawn. We could not calculate the mean HAS-BLED risk score, as not all the data points of the score components were captured in the dataset (i.e. alcohol use). The incidence of other outcomes such as stroke, mortality, discontinuation and adherence was not analyzed. Sensitivity analyses at other specific TTR thresholds (e.g. 60% or 70%) were not conducted. The stability of INR over time was not assessed. Only direct medical costs were available; these referred to all-cause costs incurred by each patient, disregarding the reason for the utilization, consequently, they could have been overestimated. Healthcare resource utilization and patient subgroups were not evaluated.

According to Brazilian standards for procedure codes (Appendix A), INR has no individual code, but it is included within the “Coagulation test” code. As it was not possible to segregate, the INR measurement was considered as the entire coagulation test, and not as a percentage of it, for all patients.

It was found that a significant portion of patients taking warfarin (11%) had CHA_2_DS_2_-VAS_C_ scores of zero, which is greater than the proportion reported in other studies (6.1%).^36^ CHA_2_DS_2_-VAS_C_ assessment is subject to the clinical documentation of patients' clinical history, and details of pre-existing conditions might have been underreported.

The phone monitoring program was offered to patients of a specific health insurance company and when a patient's contract terminated, follow-up was not possible.

Finally, some of the study limitations were inherent to a retrospective observational study design. These include potential coding errors and missing data which may have introduced biases into the study and affected the number of excluded patients, and the fact that the data assessed was not originally collected for clinical research purposes.

## Conclusions

This study examined patient profiles, quality of anticoagulation and clinical/economic outcomes among NVAF warfarin patients in a private health insurance company in Brazil. It is representative of a large and relatively young cohort of warfarin patients. The overall quality of anticoagulation management was suboptimal. Warfarin patients were within the therapeutic range slightly more than half of the time. Up to two thirds had poor control (TTR < 65%) and were associated with more bleeding events and costs. This analysis highlights the importance, in terms of outcomes and costs, of tight anticoagulation control for NVAF patients treated with warfarin, and the difficulty in maintaining an adequate TTR even with a well-designed and run program. Additional research is needed, as more real-world data becomes available, to further assess the use of warfarin as well as the adoption of NOACs *versus* warfarin.
